# Rebuilding Tendons: A Concise Review on the Potential of Dermal Fibroblasts

**DOI:** 10.3390/cells9092047

**Published:** 2020-09-08

**Authors:** Jin Chu, Ming Lu, Christian G. Pfeifer, Volker Alt, Denitsa Docheva

**Affiliations:** 1 Laboratory for Experimental Trauma Surgery, Department of Trauma Surgery, University Regensburg Medical Centre, 93053 Regensburg, Germany; chu.jin@ukr.de (J.C.); christian.pfeifer@ukr.de (C.G.P.); volker.alt@ukr.de (V.A.); 2 Department of Orthopaedic Surgery, First Affiliated Hospital of Dalian Medical University, Dalian 116023, China; dailianminglu@hotmail.com; 3 Department of Trauma Surgery, University Regensburg Medical Centre, 93053 Regensburg, Germany

**Keywords:** dermal fibroblasts, tenogenic differentiation, tendon tissue engineering, microarray data analysis, in vitro, in vivo, clinical studies

## Abstract

Tendons are vital to joint movement by connecting muscles to bones. Along with an increasing incidence of tendon injuries, tendon disorders can burden the quality of life of patients or the career of athletes. Current treatments involve surgical reconstruction and conservative therapy. Especially in the elderly population, tendon recovery requires lengthy periods and it may result in unsatisfactory outcome. Cell-mediated tendon engineering is a rapidly progressing experimental and pre-clinical field, which holds great potential for an alternative approach to established medical treatments. The selection of an appropriate cell source is critical and remains under investigation. Dermal fibroblasts exhibit multiple similarities to tendon cells, suggesting they may be a promising cell source for tendon engineering. Hence, the purpose of this review article was in brief, to compare tendon to dermis tissues, and summarize in vitro studies on tenogenic differentiation of dermal fibroblasts. Furthermore, analysis of an open source Gene Expression Omnibus (GEO) data repository was carried out, revealing great overlap in the molecular profiles of both cell types. Lastly, a summary of in vivo studies employing dermal fibroblasts in tendon repair as well as pilot clinical studies in this area is included. Altogether, dermal fibroblasts hold therapeutic potential and are attractive cells for rebuilding injured tendons.

## 1. Introduction: Tendon Injuries and Repair Strategies

Tendon and ligament ruptures are the most common entity of tendon disorders encountered in the adult population. Epidemiologic studies in the US revealed that annually 3/100,000 persons suffer from Achilles tendon rupture, 68.6/100,000 persons from Anterior Cruciate Ligament (ACL) rupture and 33/100,000 persons from acute traumatic tendon injuries of hands [1,2,3]. Tendon injuries disturb the patients’ normal work/life routine and put the career of elite athletes at risk. Furthermore, tendon healing is a slow process and easily leads to fibrotic scarring and adhesions influencing the tendon mechanical properties and function [4,5]. Current protocols to manage tendon injuries rely on non-operative therapy or on surgical reconstruction [6,7,8]. Recent studies report comparable outcomes between the nonsurgical and surgical treatment groups [9,10,11,12], but both approaches are associated with certain complications. Conservative repair has advantages in avoiding the surgery-associated complications (e.g., wound infection, scar adhesion, tendon necrosis and nerve injury), but current literature suggests a greater risk of tendon re-rupture after conservative therapy [4,9,10,13,14].

Autologous tissue grafts remain the standard of care in clinical routine for tendon and ligament replacement such as ACL reconstruction. However, harvesting autologous tendon induces donor site morbidity and disability, which limits the collection of suitable autologous tissue. Allografts and xenografts are more accessible than autografts with similar clinical outcomes, but their availability is limited and their use raises concerns such as immune rejection, possibility of disease transmission and inadequate tissue processing that can compromise the mechanical properties of the graft [14,15,16,17,18]. Thus, engineering tendon tissue, based on the combination of cells and carrier materials, has become a very attractive alternative approach for tendon and ligament reconstruction over the last decade [19,20].

In order to build up a tendon, different modes of application can be performed. One way is to inject a cell suspension (supported or not by hydrogel) directly into the site of injury [19,20,21]. Another way is to seed cells on scaffolds and cultivate in vitro to pre-engineer tendon-like tissue before implantation [19,20]. A third possibility is the implantation of decellularized tissue grafts or scaffolds alone, which can enhance the migration of endogenous immune and tendon cells to the injured tendon site. [19,20] Several cell populations have been reported to undergo tenogenic differentiation, but possess certain disadvantages [19,20]. Despite having the required attributes to generate tendon tissues, native tendon-derived cells are impacted by limitations in harvesting biopsies from uninjured tendon, which is necessary for their isolation [19,20]. Bone marrow mesenchymal stem cells (BMSCs) can induce ectopic bone formation; embryonic stem cells can be tumorigenic, whilst there are safety and efficacy concerns with reprogrammed/genetically engineered cells [20,21]. The limitations of existing cell sources and the rapidly increasing demand of cell-based tendon therapy has triggered an intense investigation in allocating an appropriate cell source for tendon tissue engineering.

In studies on mammalian skin, several stem cell populations have been identified in the dermis such as neural crest stem cells; hair follicle stem cells; mesenchymal stem cell-like dermal stem cells; and dermal hematopoietic cells [22]. These cell subpopulations were observed to possess self-renewing, multipotency as well as immune-modulating properties [22] and represent a very interesting area of research regarding applications in regenerative medicine, especially for wound healing/tissue repair, neural repair, and hematopoietic recovery. Dermal fibroblasts are another cellular component widely distributed in the dermis and easy to harvest. They exhibit multiple similarities to tendon cells in terms of morphology, gene expression and function such as collagen type I production, suggesting they may be a promising cell source for tendon engineering [23].

## 2. Review Scope and Literature Search Method

Hence, the purpose of this review article is to provide in brief information on similarities between tendon and dermis tissues and summarize in vitro studies focusing on tenogenic potential of dermal fibroblasts. Furthermore, we have carried out an analysis of open source Gene Expression Omnibus (GEO) databank (microarray of dermal fibroblasts and tendon-derived cells that can be downloaded from https://www.ncbi.nlm.nih.gov/geo/query/acc.cgi?acc=GSE3486) revealing great overlap in the molecular profiles of both cell types. This is then followed by a summary of in vivo studies employing dermal fibroblasts in tendon repair as well as pilot clinical studies in this area.

**Figure 1:**
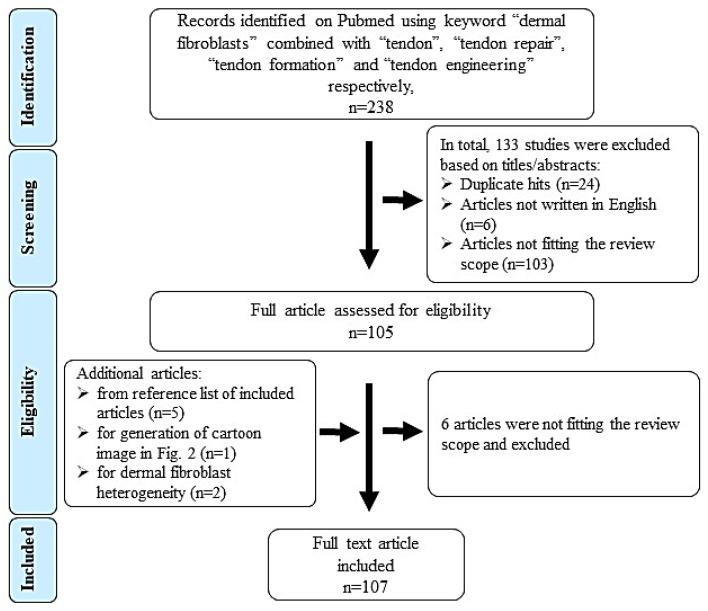
Flow chart representing the literature search and selection strategy (based on the PRISMA principle).

To identify relevant literature, we searched in Pubmed (1980 - March 2020) using the following keywords and combinations: “dermal fibroblasts” AND “tendon”; “dermal fibroblasts” AND “tendon repair”; “dermal fibroblasts” AND “tendon formation”; “dermal fibroblasts” AND “tendon engineering”. 238 hits were obtained. Figure 1 shows the literature search, selection algorithm and obtained results. 133 articles were excluded on titles/abstract level for the following reasons: duplication (*n* = 24), non-English writing (*n* = 6) and not matching or distant to the scope of this review (*n* = 103, e.g., studies focusing on skin repair not tendon, wound management and scar tissue formation).

There were 105 full text articles analyzed and at last 107 were included in this review (6 articles excluded and 8 included as they were identified in reference lists of review articles). Table 1 lists the inclusion and exclusion criteria.

It is also important to note that some of the research articles included in this review manuscript employed not only dermal fibroblasts, but also other cell types. However, for the purpose of this comprehensive review with focus on dermal fibroblasts, the other cell types were not discussed. A number of review manuscripts have focused on the comparison of different cell types for tendon tissue engineering; for example [14,19,20].

## 3. Comparison of Tissue Structure and Composition of Tendon and Dermis

Tendons are a dense connective tissue and derived from the mesoderm [24]. Unlike well-vascularized tissues such as skin, tendons are poorly vascularized and neovascularization is associated with tendon degeneration [24]. Macroscopically, healthy tendons have a white glistening appearance and consisting of packed and parallel collagen fibers. In between the well-ordered extracellular matrix (ECM), tendon-resident cells are found in rows aligned to one another. The tendon ECM is mainly composed of collagen type I (COL1) that constitutes 65–80% of tissue dry mass [19,21].

**Figure 2:**
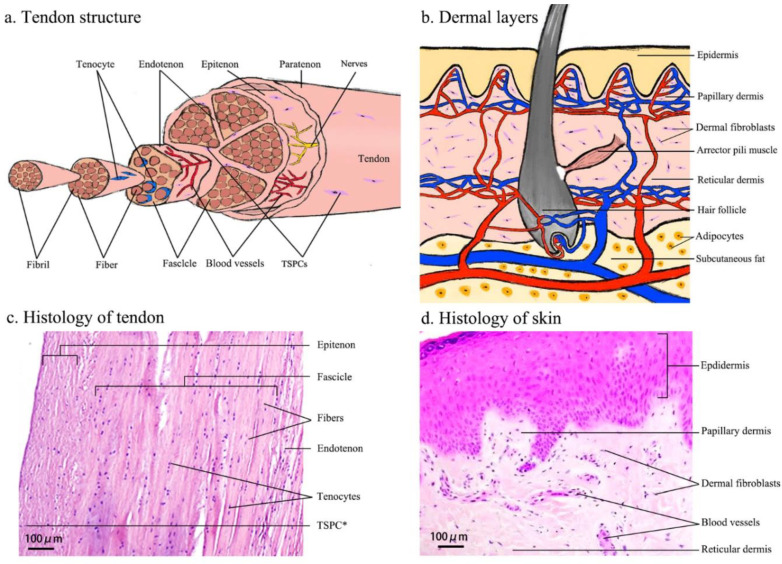
Cartooned images of the anatomical structure of human tendon (**a**) and skin (**b**), and light microscopy images of Hematoxylin & Eosin-stained tissue sections of human biceps tendon (**c**) and skin (**d**). The cartooned images were drawn by the first author and were based on [28] (for tendon) and [29] (for skin). The histological images were provided by the clinical department of the first and second authors. In (**c**) TSPC*—the star symbol indicates the putative in vivo location of TSPCs.

The basic unit of COL1 is a triple-helix tropocollagen, which is synthesized by the tenocytes (TCs) and secreted into the ECM, followed by self-assembly into parallel collagen fibrils that give the wave-like appearance of the tendon. The collagen fibrils are bundled into collagen fibers, subfascicles, fascicles and tertiary bundles that form the tendon itself and are responsible for the mechanical strength of this tissue [25,26,27]. The unique hierarchical tendon structure is illustrated in Figure 2a,c.

Tendon cells, such as TCs and tenoblasts [21], make up 90–95% of the cellular components that are essential for the production and remodeling of the ECM. Tenoblasts are progenitor, spindle-like cells with a high metabolic level and are able to transform into TCs during the tendon maturation process. TCs are specialized, terminally differentiated, spindle-like fibroblasts with a low metabolic activity that are embedded in parallel rows deeply into the collagen fibers [25,26]. Bi et al. identified an endogenous population of tendon stem/progenitor cells (TSPCs) residing within the cellular niche of hamstring tendon. The isolated TSPCs exhibit stem cell characteristics such as self-renewal, clonogenicity and the capability to differentiate into more than one specific cell type [30]. Kohler et al. [31] and Tempfer et al. [32] also demonstrated the existence of a TSPC population in human Achilles tendon and supraspinatus tendon, respectively, and both studies validated the expression of tendon- and MSC-related markers, such as Scleraxis (SCX), CD44 and nestin.

The dermis is derived from three different layers: the facial dermis and cervical dermis derive from the neural crest; limb dermis and torso dermis originate from the lateral plate mesoderm; and the dermis of the back evolves from the paraxial mesoderm [29].

Generally, the dermis consists of two sub-layers (Figure 2b,d): the papillary layer and the reticular layer. The papillary dermis is composed of loose connective tissue that is highly vascularized. The reticular layer is located beneath and is composed of dense connective tissue [33]. Collagens are the principal ECM component of the dermis. There are eighteen subtypes of collagen fibrils and eleven are present in the skin. COL1 comprises approximately 80% of the dermis ECM, whilst collagen III (COL3) comprises approximately 15% of the dermis ECM and is responsible for the pliability of the skin. The proportion of COL1 to COL3 is approximately 4:1 in unwounded adult skin, whereas the ratio is approximately 1:1 in neonatal skin. Approximately 4–5% of dermal collagen is composed of collagen V (COL5). The dermal-epidermal junction contains collagen IV (COL4) and VII (COL7), which form the structural lattice and anchoring fibrils [23,29,34].

The primary cell type in the dermis are mesenchymal dermal fibroblasts (DFs) and they are located in the papillary and reticular layers of the dermis. Interestingly, based on gene expression analyses, it is suggested that DFs are a heterogeneous population. For example, papillary dermal fibroblasts express dipeptidyl peptidase 4, netrin 1 and podoplanin, whilst reticular dermal fibroblasts are marked by the expression of CD36, actin alpha 2 and peroxisome proliferator activated receptor gamma [35]. Furthermore, Rinn et al. 2006 [36] analyzed thirty-five primary DF cultures derived from different anatomical sites of the body and found site-specific variation (anterior-posterior and proximal-distal) in their gene expression programs, suggesting DF heterogeneity is also dependent on the positional identities relative to major anatomic axes. Human DFs are capable of producing collagen, elastin and other ECM proteins. During the wound healing process, fibroblasts play an essential role in producing, remodeling and contracting the ECM of scar tissue, thus they are indispensable for the repair process [23,29,34].

## 4. Molecular Similarities between DFs and TCs: Analysis Based on Open-Source Databank

**Figure 3:**
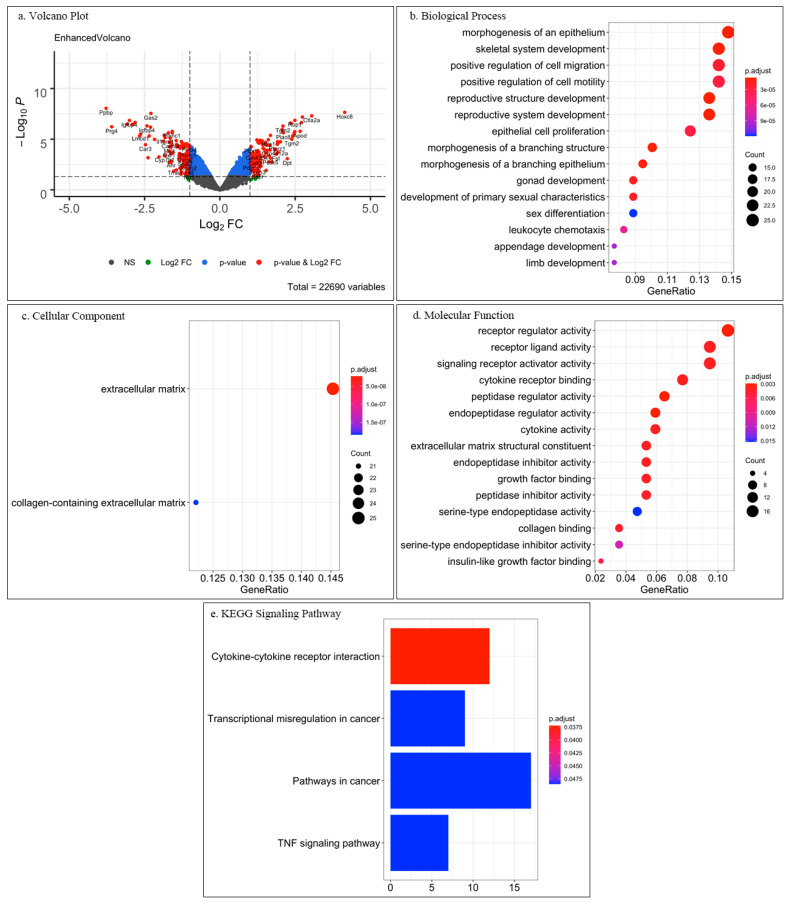
Comprehensive visualization of the major outcomes from analysis of open-source microarray data on fetal mouse dermal and tendon fibroblasts. (**a**) Volcano plot of significant differentially expressed genes (DEGs) as each gene is marked as a dot. The lines in vertical represent the Log_2_FC (fold change) value equaling 1 or −1, while the dots aside these lines are DEGs with at least 2-FC. The horizontal line shows the *p*-value of 0.05. Hence, the red dots (“*p*-value & Log_2_FC”) represented the significant DEGs with at least 2-FC; the dots in green (“Log_2_FC”) are DEGs within at least 2-FC, but are not significant; the dots in blue (“*p*-value”) have significant FC but lower than 2; and the grey dots (“NS”) are neither significant nor have 2-FC. (**b**–**d**) Gene ontology enrichment analysis of only the significant DEGs results are depicted in dot plot format where different gene clusters are listed in the *Y*-axis and the gene ratio is indicated in the *X*-axis. Top enrichment gene clusters under biological process (**b**), cellular component (**c**) and molecular function (**d**) are shown. The size of the dots represents the counts of DEGs, while the color of dots shows the significance. (**e**) KEGG signaling pathway analysis of the significant DEGs in bar plot format. The *Y*-axis shows enriched pathways, *X*-axis enrichment index, whilst the color shows the significance.

Based on the research article by Mackley et al. [37], we performed for this review a pilot analysis of the associated microarray data published in the GEO open-source databank with GEO accession GSE3486 (https://www.ncbi.nlm.nih.gov/geo/query/acc.cgi?acc=GSE3486). In this study, dermal fibroblasts (DFs) and tendon fibroblasts (TCs), from fetal mouse tissues were analyzed in parallel using Affymetrix Gene Chip mouse expression set 430 (Affymetrix, High Wycombe, UK) with the main scope to unravel cell-specific responses to mechanical stress. Furthermore, searching the GEO databank by using the keywords “dermal fibroblast tendon fibroblast microarray”, resulted in two hits (GSE5591 and GPL570). However, GSE5591 did not include a tendon fibroblast study group, and GPL570 was an introduction to Affymetrix Human Genome U133 Plus 2.0 Array and did not contain experimental data. We also checked the GEO databank with the keywords “dermal fibroblast tendon fibroblast RNA sequencing” which resulted in zero hits. For our evaluation, the microarray data GSE3486 was downloaded and we subjected it to analyse only the data of unstimulated DFs and TCs with open-source software R v3.6.3 (R Foundation for Statistical Computing, Vienna, Austria; https://www.R-project.org/) (Date of data analysis 24 June 2020). Specifically, package “GEOquery” was used for the data download from the GEO server; package “limma” was used for selecting differentially expressed genes (DEGs, genes having at least 2-fold change); package “org.Mm.eg.db” was used for gene annotation; packages “clusterProfiler” and “enrichplot” were used for enrichment analysis and visualization (volcano and dot plots). Next, Gene Ontology (GO) enriched analysis and Kyoto Encyclopedia of Genes and Genomes (KEGG) signaling pathway analysis were performed on the identified significant DEGs. In Figure 3 volcano plot of DEGs, GO and KEGG results are shown.

The obtained results revealed that among 22690 genes, only ca. 1%, namely 112 downregulated genes and 123 upregulated genes, were significant DEGs between DFs and TCs (Figure 3a). GO analysis of only the significant DEGs showed under biological process (Figure 3b), cellular component (Figure 3c), and molecular function (Figure 3d) interesting enriched clusters of gene function. For biological process, the top 15 enrichments are plotted in Figure 3b and the top five were “morphogenesis of an epithelium”, “skeletal system development”, “positive regulation of cell migration”, “positive regulation of cell motility” and “reproductive structure development”. “Morphogenesis of an epithelium” and “skeletal system development” enrichments may represent the differences between the two tissues and that DFs are strongly related to epithelium and skin development, whilst TCs relate to skeletal development.

The items referring to “cell migration” and “cell motility” might reflect wound healing and tissue repair processes. We speculate that the items “reproductive system development”, “gonad development” and “sex differentiation” appeared under biological process because the implemented cells in the study [37] were isolated from fetal tissue.

For cellular component, only two enriched items were identified: “extracellular matrix” and “collagen-containing extracellular matrix” (Figure 3c). This finding was not surprising because both dermis and tendon are mesenchymal and matrix-rich tissues. Hence, key characteristics for both cell types are ECM synthesis, deposition and remodeling.

The top 15 enrichments under molecular functions are shown in Figure 3d. and the top five were “receptor regulator activity”, “receptor ligand activity”, “signaling receptor activator activity”, “cytokine receptor binding” and “peptidase regulator activity”. This result was very interesting as it suggests that each cell type may differentially regulate cytokine and growth factor receptors as well as cell adhesion molecules. However, a closer investigation and literature follow up on target genes will be required to obtain more conclusive molecular insights and translation to cell type-specific behavior. Still, when subjecting all DEGs to KEGG analysis, yet again a major hit was “cytokine-cytokine receptor interaction” (Figure 3e). Additional hits were related to “cancer” which might be due to the embryonic nature of the DFs and TCs as well as due to links to skin cancer.

This pilot investigation demonstrated that DFs and TCs harbor great similarities at a gene expression level. However, it will be of importance to perform follow up detailed analysis of the open-source data in order to obtain predictive target genes and molecular pathways that are differentially regulated in DFs and TCs, which in turn predispose the specialized attitude of each cell type. Such and future studies, based on RNA sequencing and epigenetic analysis, can lead to the discovery of key genes and regulatory elements that can be modulated and thereby influence the DFs phenotype towards TCs.

## 5. Differentiation Potential of DFs

### 5.1. Differentiation Potential of DFs Towards Other Cell Types

Numerous studies have demonstrated that DFs are able to differentiate into other cell types [38,39]. Especially, if forced by ectopic expression of specific genes, DFs can be easily reprogrammed; for example, murine DFs differentiate into neurons under overexpression of the transcription factors Ascl1, Brn2 and Myt1l [40]. Similarly, Pang et al. converted human DFs into neurons, using the above-mentioned factors together with NeuroD1 [41]. Lmx1a and FoxA2, two transcription factors combined with Ascl1, Brn2 and Myt1l, facilitated the generation of dopaminergic neurons - a specific neuronal subtype lost in Parkinson’s disease [42,43]. By ectopically overexpressing four cardiac transcriptions factors (GATA4, Hand2, Tbx5 and Myocardin), DFs can differentiate into cardiomyocytes as well [44]. Additionally, DFs are also able to convert into multilineage blood progenitor cells and retinal pigment epithelium-like cells [45,46,47,48,49]. Collectively, the above literature points out that DFs are “naïve” cells, which under genetic modification or appropriate stimulation can be pushed towards desired cell types.

### 5.2. Tenogenic Potential of DFs

Tenogenic differentiation is a complex, step-wise process following the conversion from stem, via progenitor, to terminally differentiated, mature tendon cells. It requires the expression of tendon specific genes, secretion of ECM and its appropriate hierarchical organization, and ideally resulting in the formation of a tendon-like structure that can sustain mechanical load. So far, “a gold standard” model to mimic this process in vitro does not exist, mainly because tenogenic differentiation, similar to cartilaginous differentiation, requires combination of biochemical and biomechanical stimuli in a three-dimensional (3D) environment. Therefore, when assessing tenogenic potential of cells, the most frequent parameter monitored is validation of expression of tendon-specific genes.

DFs and TCs are of mesenchymal origin and show similarities with respect to their cell morphological appearance, transcriptome, protein synthesis of ECM including COL1, COL3 and proteoglycans, as well as capability to remodel the surrounding ECM [14]. Therefore, converting DFs and TCs can be achieved much easier than other cell types with the support of molecular, mechanical and/or biomaterial/scaffold factors. A TC-like phenotype was observed when DFs were cultured just in high density monolayer after one passage, which was validated by significant upregulation in tenogenic genes e.g., SCX, tenomodulin (TNMD), COL1, COL3, COL6, decorin (DCN) and tenascin-C (TNC) [50]. Hence, in the following sections we provide summary of in vitro, in vivo and pilot clinical studies that have pursued research into the DFs tenogenic potential in such settings.

## 6. In Vitro Studies

### 6.1. Molecular Factors

Several molecules have been determined as useful gene markers for investigating tenogenic differentiation. Tenomodulin (TNMD) is widely accepted as a mature marker of tendon and ligament lineage [51]. It belongs to the new family of type II transmembrane glycoproteins with a highly conserved cleavable C-terminal cysteine-rich domain. The gene is located on the X chromosome and accounts for a ca.14kb transcript. The predicted full-length protein is composed of 317 amino acids [52,53]. TNMD is highly expressed in tendons and ligaments, whilst low levels of mRNA transcripts have been detected in other tissues as well. A decreased proliferation of tendon cells was observed in TNMD knockout mice, resulting in a reduced TCs density and pathological thickening of collagen fibers in the tendon ECM [54]. In addition, overexpression of TNMD in murine mesenchymal stem cells (MSCs) inhibited their commitment towards the osteogenic, chondrogenic and adipogenic lineages, while promoting their tenogenic differentiation [55]. Thus, it will be interesting to investigate whether expression of TNMD can affect the phenotype of DFs in tenogenic differentiation. Liu et al. established a doxycycline-inducible TNMD overexpression mouse model. By exogenous addition of 5μM doxycycline to in vitro cultured DFs derived from such mice, TNMD was over expressed concomitantly with upregulation of several tenogenic genes (Table 2) [56].

In tendon healing, TGF-β is active at all stages of this process. Besides tendon cell migration and proliferation, all three isoforms TGF-β1, TGF-β2 and TGF-β3 increase the synthesis of COL1 and COL3 [20,21]. The production and arrangement of collagen fibers is crucial in tendon healing. Thus, TGF-β is an essential factor for the repair of injured tendons [68]. A biphasic pattern of TGF-β expression, with an early peak of TGF-β1 and a late peak of TGF-β3, was found accompanied by an enhanced expression of TGF-β receptors in flexor tendon repair in mice [69]. However, overexpression of TGF-β1 results in scar and adhesion formation of neo-tendon that leads to a decrease in mechanical strength and disability of biological function, such as joint flexibility and range of movement [70]. The up-regulated TGF-β expression can also be observed in tendinopathy and is considered to be a major predisposing factor for the development of tendinopathy. Similarly to tendon, the excessive expression of TGF-β1 can promote scarring in skin healing, whilst TGF-β3 does not induce scarring and thus has a better effect in skin wound repair and regeneration [71,72,73]. Liu et al. reported a significant upregulation of tendon-associated markers in DFs stimulated by 2 ng/mL TGF-β1. Furthermore, the authors observed that DFs could significantly enhance the endogenous production of TGF-β1, when they are to elongate by cultivation on a specially designed polymer membrane [61]. The biofunction of TGF-β1 might be concentration- and time-dependent, and cell autocrine production of TGF-β1 might be mediated by morphology-triggered intercellular tension, as well as external biomechanical stimuli. Further investigations on TGF-β signaling in DFs versus TCs will be of great relevance to understand how to steer their cellular behavior in differentiation and healing processes.

Ras homolog family member A **(**RhoA) is a small GTPase protein in the Rho family and Rho-associated protein kinase (ROCK) of the downstream molecules of RhoA pathway. The activation of RhoA/ROCK signaling pathway has been implicated in various biological processes including stress fiber formation, remodeling of ECM and inhibition of chondrogenic and osteogenic differentiation processes. Blocking of RhoA/ROCK signaling pathway resulted in a loss of elongated morphology of human MSCs and a decreased expression of tenogenic marker genes [74,75,76,77,78]. Interestingly, in tissue aging, TSPCs start to express abnormal levels of ROCK, accompanied by enlarged polygonal cell morphologies, and when ROCK activity was reduced by kinase inhibitor, aged TSPCs rejuvenated to a large extent [31]. Furthermore, morphological changes induced by subjecting cells to tension can be reversed by blocking ROCK activity. Xu et al. evaluated the effect of ROCK inhibitor on tenogenic gene expression in human MSCs that were stretched and detected significant decreases in the expression of SCX, COL1 and COL3 [76,77]. Based on the above data, it will be of great interest to study the role of ROCK in DFs in the context of tenogenic differentiation. In order to understand the discrete steps of tenogenic differentiation, it will also be important to better characterize the molecular signature of TSPCs, tenoblasts versus TCs, which can lead to identification of regulatory molecules. This can be then used to elegantly nurture DFs’ tenogenic potential.

### 6.2. Mechanical Factors

The main function of tendon is to transfer tensile loads from muscle to bones. Thus, mechanical stimulation should be an important factor for tenogenic differentiation and maturation. Several studies revealed that static mechanical strain enhances tendon-associated gene expression in DFs [37,58,66]. Moreover, dynamic strain was even more effective in DFs tenogenic differentiation [64,79]. Interestingly, rat bone marrow cells exhibited osteogenic potential when they were cultured on flexible dishes and subjected to 1 Hz deformation cycles; however, no such effect was seen in DFs [79]. Additionally, an investigation of tension-induced tenogenic differentiation of TCs showed a clear downregulation of α11 integrin receptor subunit at the mRNA and protein levels, but an upregulation of α2 subunit when tension was omitted [80], suggesting an interesting integrin switch in the mechanotransduction process.

Another tension-loaded model with DFs revealed deposition of longitudinally aligned collagen fibers and spindle-shaped cells. This model also showed a significantly improved in vitro tendon-maturation score when compared with a tension-free cultivated DFs [66]. Di et al. observed more aligned cell arrangement and collagen in mechanically stimulated tendons versus unstimulated ones [64]. Mackley et al. stimulated fetal mouse DFs, tendon and cornea fibroblasts by mechanical force and microarray analysis showed that the expression of 344 genes was significantly changed, with 8% of the altered genes being the same in tendons and dermis and 7% being unique to DFs. Follow up on the function and relevance of mechano-sensitive candidate genes in tenogenic differentiation of DFs will be of great interest [37]. Altogether, mechanical stimulation of DF holds promising potential to steer their fate towards TC (Table 2); however, the translation to biochemical pathways should be better understood in future.

### 6.3. Cultivation Environment

A series of studies have focused on investigating how cultivation environment, e.g., high density culture, macro-molecular crowding, or different biomaterials influence the behavior of DFs in the context of tenogenesis (Table 2). Two studies by Liu et al. [50,61] have analyzed whether tenogenic genes and protein expression can be influenced by culture modifications. First, they found that high density cultivation induces a significant up-regulation of only tendon-associated gene expression in DFs, but not in genes related to osteogenic, chondrogenic and adipogenic cell lineages. They also observed that the gene expression levels for SCX, TNMD, COL1 and DCN were remarkably downregulated after switching the cultivation density from high to low, indicating that tenogenic differentiation process might be cell density-dependent. In the second study, the authors discovered that tenogenic differentiation of DFs is also affected by cell morphology. By growing DFs on a silicone membrane with microgrooves on its surface, the cells were forced into a more elongated cell shape which was accompanied by enhanced production of tendon-associated proteins. However, yet again there were no significant changes in genes typical for chondrogenic and osteogenic differentiation.

In order to create a 3D microenvironment in vitro, numerous polymers, natural and synthetic, have been assessed in tendon engineering [81,82]. Collagen-based materials, a natural polymer, allow fabrication of 3D scaffolds that are close to the native tendon tissue environment, due to the fact that collagen is the most abundant component of the tendon ECM [83,84]. Collagen sponges show promise for improving tendon regeneration, with or without fiber alignment and additional cell co-application [85,86,87]. Furthermore, it has been suggested that collagen-based materials not only facilitate bidirectional cell elongation, but can also maintain tenogenic phenotype in vitro [88,89].

Silk-based materials have been widely used in suturing ruptured tendons on account of their high mechanical properties and biodegradability [90,91]. In vitro studies have revealed that silk-based scaffolds are suitable for adherence, expansion and differentiation of human MSCs towards the tenogenic lineage, which was evaluated by the expression of COL1, COL3 and TNC genes [92].

Synthetic polymers have also been extensively evaluated in tendon repair, and can be produced with specific structural, physiological and mechanical properties similar to the tissue in need of replacement [82,93]. Polyglycolic acid (PGA)-based scaffold, appears to be an alternative material in tendon engineering, and aligned orientations of the PGA fibers are found to be more effective in maturation of neo-tendon [63]. Polylactic acid (PLA)-based scaffolds or combined with silk scaffolds have been assessed for tenogenic differentiation of DFs and have also demonstrated good results [65,94]. Hallie et al. investigated fetal and adult rat DFs cultivated with or without a synthetic scaffold. Interestingly, fetal DFs had superior tenogenic potential than their adult counterparts [59]. However, the study did not discuss differences between scaffold and non-scaffold groups. Jiang et al. performed an interesting study with acellularized DFs-derived matrix (dFM). To produce the matrix, DFs were cultivated in six-well plates for 3 weeks and then removed. Next, the dFM was seeded with tendon stem cells (TSCs) and it was observed that they grew faster than on plastic surface. Moreover, TSCs significantly upregulated genes such as TNMD, COL1 and SCX [60].

Another area of investigation is the pattern fiber organization in scaffolds. There is evidence suggesting that the parallel alignment of the fibers improves the expression of tendon-related genes. Chen et al. cultivated DFs in aligned versus random nano-composite scaffold, and reported a significant improvement in tenogenic-associated genes [65]. Thus, the macro- and nano-topography of the scaffolds can serve as a guiding factor for tenogenic differentiation of DFs. It is well possible that the macro- and nano-biomechanical properties, such as material stiffness are also very influential; therefore, future studies along these lines should be encouraged.

In the last decade, many promising results were obtained; however certain issues still have to be overcome. For example, natural scaffolds require additional treatment to avoid in vivo immunoreaction, frequently leading to compromised mechanical properties. Synthetic scaffolds are associated with concerns on low cell adhesion, unsatisfactory cell proliferation and differentiation, poor biocompatibility and low biodegradability [20]. Hard scaffolds need more complicated surgery to be implanted into the injury site than hydrogels, which permit minimally invasive long-distance delivery. A three-dimensional cell sheet model, which is a scaffold-free tendon-like tissue, could be a powerful new approach for tendon tissue engineering. Such a cell sheet model does not need natural or synthetic carriers, avoiding the disadvantages listed above. This model allows cells forming up a continuous integral cell layer, dispositioning own native ECM and creating a 3D tendon-like tube, which can be centimeters long and subjected to static or dynamic tension for a desired period of time. Our previous study proved that human TSPCs have great potential to form well-aligned and collagen-rich cell sheets [95]. Moreover, our further research revealed that aged/degenerative TSPCs exhibit defective cell sheets compared to young/healthy TSPCs [96], demonstrating that the cell sheet model is suitable to mimic in vitro tenogenic differentiation process as well as to investigate pathomechanisms involved in tendon aging. Hence, it will be of great interest to perform a follow up study with the aim to subject DFs to the tendon cell sheet protocol.

The investigations on tenogenic potential of DFs in vitro pointed out several intriguing molecules, but the associated signaling pathways remain by and large unclear. Mechanical stimulation, cultivation environment and certain scaffold materials can enhance DF tenogenic differentiation, but yet again the underlying mechanisms and efficacy of the process are to be clarified. As mentioned above, one issue at present is that tenogenic differentiations is in most cases concluded only on the mRNA expression levels of few genes. Hence, developing “true” tenogenic differentiation protocols in vitro and adequate methods to monitor the step-wise conversion of the cells will be of great value for this research field. Studies focusing on uncovering regulatory molecules accelerating tenogenesis should also be highlighted in future.

## 7. In Vivo Studies 

Animal models offer an essential framework for studying tissue physiology and pathology as well as regeneration in many fields. Along with the loss of continuous force transmission from skeletal muscles, scientists observed in animal models a reduction in tendon size and impaired tendon biomechanical properties [37,64,65,80,97]. Appropriate animal models allow scientists to understand molecular, cellular and tissue-level principles underlying tendon injury and repair, and they can also promote the development of new therapeutic strategies [98,99]. Bottagisio et al. indicated that rabbits (49%) are the most commonly used models followed by rats (27%) and mice (6%) when studying tenotomy repair. Large animal models are also employed in preclinical studies for the same purpose [98]. Despite the advantages of lower cost and easier handling of medium and small animal models, large animals (canine, ovine, bovine, pig, etc.) overcome certain limitations related to tendon size, hormone patterns, loading regime, and clinical translation, as they have more similar characteristics to human tendon [99,100].

In vivo studies are very adequate to evaluate biochemical, biomechanical and overall biocompatible properties of scaffolds (summarized in Table 3).

Liu et al. established a pig flexor tendon defect model and implanted PGA scaffolds seeded with autologous DFs. Biomechanical analysis at 26 weeks post-operation demonstrated that the tensile strength of DF-engineered tendon reached 31.0 ± 2.2 MPa, 74% of normal tendon. Meanwhile, the tensile strength of TC-engineered tendon reached 32.4 ± 3.6 MPa, approximately 76% of normal tendon property. This result suggested that DF- and TC-engineered tendons achieve similar mechanical properties and could reconstitute normal tendon function, and thus are suitable for replacement of injured tendons [102].

A PGA/PLA scaffold co-implanted with DFs, was employed to evaluate the tenogenic potential of autologous and allogeneic DFs. The results demonstrated a more mature tendon-like tissue in the autologous cell variant compared to allogeneic cells or without cells [94]. Another study indicated that the scaffolds with parallel aligned nanofibers can induce appropriate cell orientation, as well as DFs differentiation towards TCs, leading to the secretion of ECM typical for tendon tissue [65].

Based on the promising outcomes in literature, additional pre-clinical studies involving DFs combined with different carriers will be of great relevance to carefully evaluate the clinically translational power of this cell type in restoring tendons. It will be very appreciable in future in vivo studies to include in-depth validation of the survival and differentiation of the implanted DFs by their tracking at different time points, whether there is a crosstalk to immune and endogenous TCs, scar properties, adhesion formation, biomechanical properties as well as long-term functional assessment such as gait and endurance analyses.

## 8. Clinical Applications of Autologous DFs in Tendon Repair

Of course, the ultimate goal of DFs research in the context of tendon regeneration is translation to clinical application for improving patient tendon function and patient quality of life. We identified two frontier studies in this area. Connell et al. reported a pilot clinical study and a randomized controlled trial in 2009 and 2010, respectively [105,106] (Table 4).

In the pilot study, twelve patients were diagnosed with refractory lateral epicondylitis. Patient-Rated Tennis Elbow Evaluation (PRTEE) scale and ultrasonography were used as measurements to evaluate the level of injury as well as recovery. Laboratory-prepared collagen-producing cells derived from dermis were used as a cell therapy. There was a significant PRTEE score improvement compared to pre-treatment values and at each of the subsequent evaluation time points. Ultrasonography examination showed a tendency for better tendon appearance in terms of decrease in tendon size and restoration of the fibrillar pattern as well as a near-total resolution of the intrasubstance tears following the administration of autologous DFs. It is worth mentioning that out of the twelve patients, eleven patients reported a satisfactory outcome, and only one underwent surgery at three months post-treatment with cells.

One randomized controlled trial included 46 patients (60 patellar tendons were treated) with refractory patellar tendinopathy. Victoria Institute of Sport Assessment (VISA) score and sonographic examination were used as measurement methods to evaluate level of injury and recovery. Cell therapy as an intervention consisted of autologous plasma with/without laboratory-prepared amplified collagen-producing cells derived from dermis. The authors concluded that ultrasound-guided injection of autologous DFs can be safely used, in the short term, to treat patellar tendinopathy, with good tissue response to treatment and a significant improvement in pain and function compared to plasma alone [105]. The authors annotated the cells used in their studies as laboratory-prepared amplified collagen-producing cells derived from dermis, but the cells were not validated at the molecular level as DFs. To gain higher reproducible value, establishing protocols for cell type characterization of the implemented cells would be required. This is also an important base for developing standardized medicinal products for clinical application. To our knowledge, there are currently no registered trials using DFs implantation for tendon therapy [107]. However, we believe that if the field gains new breakthroughs in the understanding of DFs’ tenogenic potential by using tendon in vitro and in vivo models, such trials could be initiated in the future.

## 9. Conclusions and Outlook

Various studies have already suggested that DFs harbor tenogenic potential in vitro and in vivo as well as pilot clinical trials have been performed. This valuable body of work has set up the foundations for using DFs as therapeutic cell source to rebuild tendons. Still, there is enough space for further investigations and improvements, which can provide answers to many open questions. For example, more study is needed on developing in vitro tendon differentiation model resembling more closely the critical assets of in vivo tenogenesis; ideally, also being user-friendly and independently validated in different research groups, will allow cross comparison between study results, as well as better reproducibility of findings. Identifying molecular factors that are capable of inducing DFs to form mature tendon tissue and/or stable tendon tissue phenotype would be of great interest. So far, most cell-scaffold constructs match poorly the mechanical properties to native tendon tissue. Hence, there is great room for discoveries on how micro- and nano-topography and biomechanical properties of biomaterials can steer or support the fate decision of cells. Scalability of 3D-engineered tendon-like tissue to the dimensions and strength of human tendons should be addressed, as well as how their integration in vivo at the site of injury can be improved. Last but not least, execution of more clinically relevant large animal models should be highlighted as an essential step prior to developing novel clinical applications. Altogether, research efforts along the above lines can result in “a step forward” in cell-based therapy of injured tendons.

## Figures and Tables

**Table 1:** Inclusion and exclusion criteria.

Inclusion Criteria	Exclusion Criteria
**Title/abstract**	
Studies reported in English. Studies focusing on dermal fibroblasts in the context of tendon repair. Studies focusing on tenogenic differentiation of dermal fibroblasts. Studies assessing tenogenic properties of new scaffold materials using dermal fibroblasts.	Duplicates. Non-English text. Studies focusing on dermal fibroblasts in the context of skin tissue. Studies focusing on tenogenic differentiation but without data on dermal fibroblasts. Studies assessing tenogenic properties of new scaffold materials without using dermal fibroblasts.
**Full text**	
In vitro studies. In vivo studies. Clinical studies. Reviews on tissue composition of skin and tendon.	Studies focusing on dermal fibroblasts but no relation to tendon tissue. Studies focusing on tendon tissue but no relation to dermal fibroblasts.
	Studies focusing on skin wound management.
	Studies focusing on skin scar tissue formation.

**Table 2:** In vitro studies on tenogenic potential of DFs (in chronological order).

Species	Material of Scaffold	Stimulation Factors	Results	Ref.
Mice	-	Static mechanical strain	Microarray analysis revealed 344 DEGs upon stimulation. Skin and tendon fibroblasts shared 8% of DEGs, while 7% of DEGs were unique to the fibroblasts derived from the skin.	[37]
Rabbit	Acellularized tendon	-	There were no significant differences among native, acellular and DFs-seeded tendons in comparing ultimate loading force. DFs-seeded tendons became significantly elongated compared with native and acellular tendons in load-to-failure test. No obvious changes in ECM molecules (COL1, Pro-COL1, COL3, COL4, COL6, versican, vimentin) were observed after acellularization of the tendon. The seeded DFs could synthesize pro-collagen I after cultivation on the acellularized tendon.	[57]
Human	PGA	Static mechanical strain	Human tendon-like tissue can be generated with human DFs and PGA fibers under static mechanical strain. No difference was detected between human DFs and TCs engineered neo-tendons with regards to their gross appearance, histologic structure, collagen fiber organization and mechanical properties.	[58]
Rat	PGA	-	In monolayer culture, fetal DFs produced significantly more COL1 and COL3, displayed serum-independent growth while adult DFs elaborated less collagen, and exhibited reduced cell spreading and attachment under low-serum conditions. In 3D culture, fetal constructs contained significantly more total DNA and protein as well as deposited COL1 to adult counterparts. After 35 days, fetal constructs possessed superior mechanical properties compared to the adult ones.	[59]
Rat	dFM	-	The TSCs grew faster on dFM than that on plastic surface. COL1, TNMD and SCX significantly increased in TSCs after seeding onto dFM.	[60]
Human	-	High density culture	The gene expression levels for SCX, TNMD, COL3 and decorin were remarkably downregulated in DFs after switching the cell density from high to low. High density culture could enhance the expression of TGF-β1, GDF5, 6, 8 but not GDF7 in DFs. TGF-β1 inhibitor could significantly inhibit high density-induced gene expression of SCX, TNMD, COL1, COL3 and TNC but did not affect COL6 and DCN.	[50]
Human	Grooved silicone membrane	Morphology, exogenous TGF-β1 and Rock inhibitor	Enhanced expression of SCX, TNMD, COL1, COL3, COL6 and DCN was observed in elongating DFs but chondrogenic (COL2 and aggrecan) and adipogenic (PPAR-γ, CCAAT/enhancer binding protein-α, activating enhancer binding protein-2α) markers were not influenced. Osteogenic markers (alkaline phosphatase and osteocalcin) were significantly downregulated. Elongated cell morphology enhanced TGF-β1 expression and ROCK activity. 2 ng/mL of exogenous TGF-β1 treatment could significantly enhance the gene expression of SCX and TNMD. Reducing ROCK activity resulted in downregulation of tenogenic gene markers in elongated DFs.	[61]
Human	Native hydrogel	Cell density	Tendon tissue extracted hydrogel induces tenogenesis (SCX, TNC, COL1, COL3 and PLOD2) of DFs and ADSCs seeded at low density (0.5 million cells/mL).	[62]
Human	PCL/gelatin nanofiber	Pattern of nanofibers	The well-aligned nanofibers enforced hDFs to elongate, induce a tenogenic-like phenotype and form better organized neo-tendon in comparison to random nanofibers. A significant upregulation of tendon-related genes (SCX, Mohawk Homeobox, TNMD, COL1, COL6, TNC, biglycan and fibromodulin) was observed, at day 3 and 7 post-cell seeding, in DFs on aligned nanofibers than on random.	[63]
Pig	Collagen I	Static and dynamic mechanical strain	The fibers organized along the direction of the mechanic force. DFs and ASCs secreted higher COL3 than COL1 when compared to TCs and native tendon tissue.	[64]
Mice	-	TNMD overexpression	Upon TNMD overexpression SCX, COL1, COL3, COL6, DCN and TNC upregulated. DFs exhibited a tenogenic potential better than ADSCs.	[56]
Rabbit	PCL/Silk Fibroin Nanofiber	Aligned vs. not-aligned nanofibers	A significantly higher expression of tenogenic genes (COL1, fibronectin and biglycan) was observed in the aligned nano-composite scaffolds.	[65]
Human	BIO-3D printed tissue ring	Static mechanical strain	The group subjected to tension revealed longitudinaly aligned collagen fibers, which with prolonged culture increased in numbers as well as more spindle-shaped DFs were observed. The “tension” group showed a significantly improved in vitro tendon-maturing score in comparison to the “tension-free” group. Immunohistochemistry revealed parallel to the tensile direction TNC arrangement as well as stronger SCX staining than the group without tension.	[66]
Human	-	MMC and mechanical stimulation	Perpendicular to the load alignment was observed with TCs, DFs, BMSCs and ADSCs. MMC did not affect cell orientation. All cell sources exhibited enhanced deposition of COL1A1 under MMC.	[67]

Abbreviations: ADSCs = adipose-derived stem cells, DEGs = differentially expressed genes, dFM = de-cellularized dermal fibroblast-derived matrix, GDF = growth and differentiation factors, MMC = macromolecular crowding, PCL = Polycaprolactone, PGA = polyglycolic acid, TGF-β = transforming growth factor-β, TSCs = tendon stem cells.

**Table 3:** In vivo studies on tenogenic potential of DFs (in chronological order).

Model	Cell Type	Scaffold Type	Implants Classification	Results	Ref.
Rabbit Achilles tendon defect	Rabbit DFs	HA-ECM	Scaffold with DFs Scaffold alone Just suture	Parallel fibers and oval cells were observed in immunocytochemistry staining 2 and 3 months post operatively. After 3 months of operation, biomechanical tests revealed that the tensile strength of neo-tendon with DFs could achieve 81.8% of that of normal tendon.	[101]
Pig flexor tendon defect	Pig DFs	PGA	Scaffold with DFs Scaffold with TCs Scaffold alone	DFs and TCs engineered tendons were similar to each other in their gross view, histology, and tensile strength. DFs and TCs engineered tendons did not express COL3 suggesting a phenotype shift of DFs onto PGA which normally produce high levels of COL3. The scaffold alone group was histologically disorganized and mechanically weaker than both cell-engineered tendons.	[102]
Rat patellar tendon injury	TSCs	dFM	TSCs with dFM TSCs alone	COL1 fiber alignment was improved in the dFM and TSCs group. The ultimate stress of the patellar tendons was significantly higher in dFM and TSCs group than in TSCs only group.	[60]
Rat subcutaneous pocket	DFs, TCs and MDCs	PGA	Scaffold with DFs Scaffold with TCs Scaffold with MDCs	A higher level of collagen maturation in MDC–engineered tendon versus DF and TC groups dermal fibroblast and tenocyte groups. Tensile strength of the MDC group was significantly higher than the other two groups.	[103]
Rat subcutaneous pocket	DFs, ADSCs	Tendon tissue derived hydrogel	Scaffold with DFs Scaffold with ADSCs	A greater number of DFs could be detected int the tendon tissue-derived hydrogelcompared with ADSCs.	[62]
Rabbit Achilles tendon defect	DFs	PGA and PLA composed scaffold	Scaffold with autologous DFs Scaffold with allogeneic DFs Scaffold alone	Demonstrates the feasibility of autologous, allogeneic and cell-free scaffold approaches in tendon regeneration. However, the autologous scaffold formed relatively more mature (histological scoring based on H&E staining) tendon-like tissue on 13^th^ month of post-surgery.	[94]
Rabbit Achilles tendon partial defect	Rabbit DFs	PLC/Silk Fibroin nanofiber scaffolds	Acellular RPSF Acellular APSF Cells/RPSF Cells/APSF	The amount and the orientation of ECM deposition of rabbit DFs was enhanced by the aligned nanofibers. APSF induced oriented arrangement of cells and differentiation of rabbit DFs to TCs. There was no significant difference in maximum load between cells/APSF and normal tendon.	[65]
Rabbit rotator cuff tear	DFs	-	DFs suspension with fibrin Fibrin only Saline only	Demonstrated the potential for DFs to promote tendon-to-bone healing in terms of histological biomechanical outcome.	[104]

Abbreviations: APSF= aligned PCL/Silk Fibroin nanofibers, HA-ECM= human amnion ECM, RPSF= random PCL/Silk Fibroin nanofibers, MDCs = muscle-derived cells.

**Table 4:** Clinical application in tendon reparation (in chronological order).

Clinical Diagnosis	Study Design	Interventions	Results	Ref.
Refractory lateral epicondylitis	Prospective clinical pilot study.	Autologous cells suspended in autologous plasma.	PRTEE score was significantly improved after intervention compered to pre-treatment values. Ultrasonography examination showed a tendency for healthier tendon appearance (decrease in tendon size, restoration of the fibrillar pattern) and resolution of the intrasubstance tears upon intervention. 11 of the 12 patients, reported a satisfactory outcome. Only one patient required surgical treatment after 3 months.	[106]
Refractory patellar tendinopathy	Randomizedcontrol trial	Autologous cells suspended in autologous plasma. Autologous plasma alone.	A significantly improvement in VISA score was observed in the cell group. This group also reported significantly faster improvement of symptoms. A significant positive effect of the treatment was also detected in sonographic evaluation of the patella.	[105]

Abbreviations: PRTEE = Patient-Rated Tennis Elbow Evaluation, VISA = Victoria Institute of Sport Assessment.
